# A flexible protruding microelectrode array for neural interfacing in bioelectronic medicine

**DOI:** 10.1038/s41378-022-00466-z

**Published:** 2022-12-22

**Authors:** Helen Steins, Michael Mierzejewski, Lisa Brauns, Angelika Stumpf, Alina Kohler, Gerhard Heusel, Andrea Corna, Thoralf Herrmann, Peter D. Jones, Günther Zeck, Rene von Metzen, Thomas Stieglitz

**Affiliations:** 1 NMI Natural and Medical Sciences Institute at the University of Tübingen, Reutlingen, Germany; 2 Laboratory for Biomedical Microtechnology, Department of Microsystems Engineering, University of Freiburg, Freiburg, Germany; 3 Institute of Biomedical Electronics, TU Wien, Vienna, Austria; 4 Bernstein Center Freiburg, University of Freiburg, Freiburg, Germany; 5 BrainLinks-BrainTools, University of Freiburg, Freiburg, Germany

**Keywords:** Engineering, Materials science

## Abstract

Recording neural signals from delicate autonomic nerves is a challenging task that requires the development of a low-invasive neural interface with highly selective, micrometer-sized electrodes. This paper reports on the development of a three-dimensional (3D) protruding thin-film microelectrode array (MEA), which is intended to be used for recording low-amplitude neural signals from pelvic nervous structures by penetrating the nerves transversely to reduce the distance to the axons. Cylindrical gold pillars (Ø 20 or 50 µm, ~60 µm height) were fabricated on a micromachined polyimide substrate in an electroplating process. Their sidewalls were insulated with parylene C, and their tips were optionally modified by wet etching and/or the application of a titanium nitride (TiN) coating. The microelectrodes modified by these combined techniques exhibited low impedances (~7 kΩ at 1 kHz for Ø 50 µm microelectrode with the exposed surface area of ~5000 µm²) and low intrinsic noise levels. Their functionalities were evaluated in an ex vivo pilot study with mouse retinae, in which spontaneous neuronal spikes were recorded with amplitudes of up to 66 µV. This novel process strategy for fabricating flexible, 3D neural interfaces with low-impedance microelectrodes has the potential to selectively record neural signals from not only delicate structures such as retinal cells but also autonomic nerves with improved signal quality to study neural circuits and develop stimulation strategies in bioelectronic medicine, e.g., for the control of vital digestive functions.

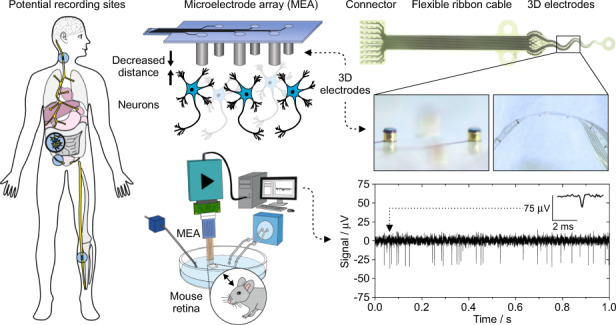

## Introduction

**Fig. 1:**
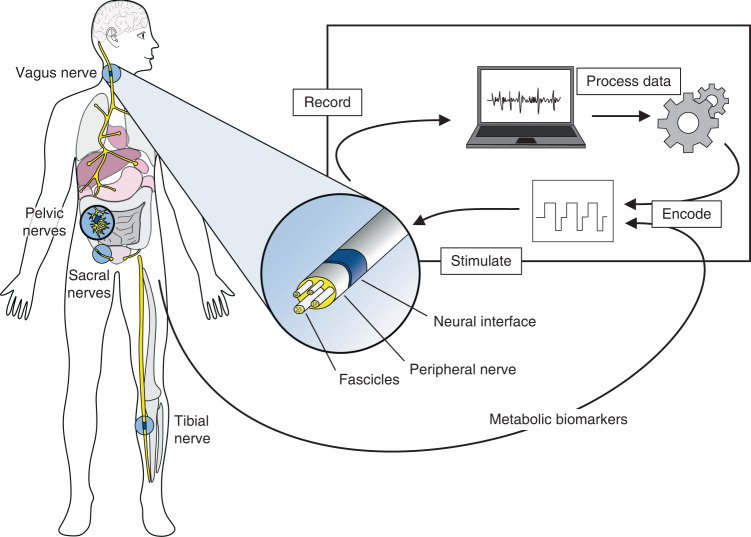
Schematic principle of bioelectronic medicine. Implanted electrodes address a variety of peripheral nerves to record neurophysiological signals. The data are decoded and processed to specify appropriate stimulation patterns and accurate dosage to modulate pathological signals. Moreover, metabolic biomarkers are obtained from the body to provide feedback, creating a closed-loop system for the automatically controlled regulation of organ function. In our approach, we aim to address pelvic neural structures with a flexible, protruding microelectrode array

In recent years, bioelectronic medicine has gained increasing attention as a therapeutic approach for the treatment of various neuronal disorders and dysfunctions^1^. Since the language of the body is electrical in nature, it seems reasonable to use electricity to specifically target neuronal circuits, read out the electrical information and modulate pathological neural miscommunication by writing in electrical stimulation patterns. This bioelectronic therapeutic approach (Fig. 1), combined with advances in the technology development of electrical microimplants, introduces a revolutionary new treatment method that appears to be superior to conventional medication due to its specificity^2^.

With the ability to electrically address both the central and peripheral nervous systems of the body, neural interfaces have been shown to be promising treatment tools for a wide range of applications^3^. While much research has been conducted on the development of cortical interfaces^4–6^, neural sensory and motor prostheses^7–10^ and vagus nerve interfaces^11–13^, there are only a few approaches for electrically addressing autonomous nerve fibers of the enteric nervous system.

Autonomic body processes, such as peristalsis or digestive and metabolic processes, are regulated by small pelvic nerves, which have highly branched and complex structures^14^. The pathological disorders and dysfunctions of these neuronal regulation circuits directly affect the motility of digestive organs, which in turn can result in chronic diseases, such as constipation, fecal incontinence or urinary dysfunction^15^. These diseases are conventionally treated with medication or by surgical interventions without taking the cause of the disease into consideration^16^.

To effectively treat neural disorders of the autonomic nervous system, it is crucial to identify and map pathological neuronal signaling chains and subsequently modulate them on the basis of healthy signals in a closed-loop manner^17^.

Selectively recording signals from specific autonomic nerves is a real challenge, as the sizes of these nerves are often in the submillimeter range^18^ and supportive layers surround the nerve (epineurium), the nerve fascicles (perineurium) and the axons (endoneurium)^19^. Autonomic nerves are mainly dominated by unmyelinated C-fibers^20^ generating low-amplitude waveforms^21^ with conduction velocities below 1–2 m/s^22,23^.

To address these nerves, the development of innovative, highly specific and low-invasive neural interfaces with micrometer-sized electrodes for forming technical-biological interfaces is needed.

Reducing electrode impedance is an important goal in manufacturing, as electrode impedance scales with thermal noise, which is theoretically the physical contribution to the total noise of the measuring electrode. Recording with low-impedance electrodes can therefore improve the signal-to-noise ratio (SNR), which in turn makes it possible to measure lower signal amplitudes^24–27^. The electrical impedance of an electrode can be lowered by increasing the electrode size. However, as a consequence, the electrode becomes less selective, as the measured neural signals are averaged over its surface area^26,28^.

A second way to decrease electrode impedance is to increase the electrode’s active electrochemical surface area by coating it with additional materials (e.g., titanium nitride (TiN) or iridium oxide (IrO_x_))^29–31^ while maintaining the same electrode diameter^32^. By modifying the electrode surface, it is thus possible to produce small electrodes with a low electrical impedance that allow more selective measurements with high SNRs.

Additionally, the use of microscopic electrodes with refined electrode surfaces offers the possibility of increasing the number of electrodes per area. This results in higher spatial resolution during recording and stimulation^28^.

The distance between the electrode and signal source is another important factor that defines the SNR. Placing the electrodes close to the target structures significantly improves the signal quality^19,33^. In this context, nerve electrodes can be classified as extraneural or intraneural with respect to their location in regard to the epineurium^34,35^.

The most commonly used extraneural electrode for interfacing with peripheral nerves is the cuff electrode^19,36^. Since cuff electrodes do not penetrate the epineurium, they have a low degree of invasiveness^35^, which has a positive effect on their longevity. However, being spatially further away from the signal sources, they measure the overall nerve activity instead of single-unit activities^37^.

Intraneural electrodes are placed inside a peripheral nerve and allow the recording of neural activity with increased selectivity and higher SNRs from specific fascicles (interfascicular) or even axons (intrafascicular).

The longitudinal intrafascicular electrode (LIFE)^38^ and the transverse intrafascicular multichannel electrode (TIME)^39^ are prominent examples of intrafascicular electrodes. While the LIFE is implanted as a fine wire in a thread-like manner parallel to nerve fibers and is usually in contact with a single fascicle^40^, the TIME is placed perpendicularly through the nerve, addressing one or even several fascicles simultaneously. The LIFE and TIME are mainly used for neuroprosthetic applications^41–43^ targeting single nerves. However, due to their two-dimensional (2D) nature, they address only a certain number of fascicles. For this reason, Thota et al. developed an array of LIFEs, denoted as the distributed intrafascicular multielectrode (DIME), which consists of several (six) LIFEs to increase the recording area^44^. Nevertheless, the placement of the device is complicated and time-consuming and, thus, ultimately limits the electrode number.

The Utah slanted electrode array (USEA) is a three-dimensional (3D) neural interface belonging to the third category of intrafascicular electrodes. It consists of an array of electrodes with varying heights allowing the simultaneous recording and stimulation of several fascicles^45,46^. Originally developed as an intracortical neural interface^45^, the USEA has additionally found application in other areas, e.g., for bladder control^47,48^.

Mathews et al. used a high-density (HD) USEA (48 electrodes, 30–100 µm tapered diameter, pitch 200 µm) to record signals from feline pudendal nerves, verifying that the USEA can be used to record signals from large (~1 mm) autonomic nerves^17^. However, the silicon-based HD-USEA is most likely too rigid, with electrodes too large for chronic recording from small-diameter (<0.5 mm) autonomic nerves^49^.

Because the fabrication process of the USEA is based on wafer-etching, conventional 3D microelectrode arrays (3D MEAs) are based on rigid silicon as the substrate material^50^. However, Lee et al.^51^ and Yan et al.^52^ demonstrated the possibility of producing flexible Utah-like 3D MEAs with silicon pillars by combining several micromachining techniques.

For interfacing with the peripheral nervous system (PNS), flexible polymers, such as polyimide (PI), parylene C, polydimethylsiloxane (PDMS), or liquid crystal polymer (LCP), are essential as substrate materials to match the mechanical properties of the surrounding tissue^53,54^.

A combination of the 3D MEA approach with flexible polymer materials and electrode modification processes could enable improved neural interfaces for recording small-amplitude neural signals from autonomic nerves. Expanding the spectrum of fabrication methods capable of producing protruding electrodes is promising for the future of bioelectronic medicine.

In this paper, we present a novel technological approach for fabricating a 3D MEA with 32 cylindrical microelectrodes arranged on a flexible PI substrate. Individual recording sites are located at the tips of high aspect ratio pillars. The pillars exhibit a sidewall parylene C passivation layer. Their heads were selectively modified by different micromachining processes to decrease their impedance properties. In vitro characterization, as well as ex vivo system validation, proved the presented concept. The developed flexible 3D MEA thus represents an interesting tool for recording neuronal activity with high SNRs from pelvic neural structures.

## Materials and methods

### Design of the flexible 3D microelectrode array (3D MEA)

**Fig. 2:**
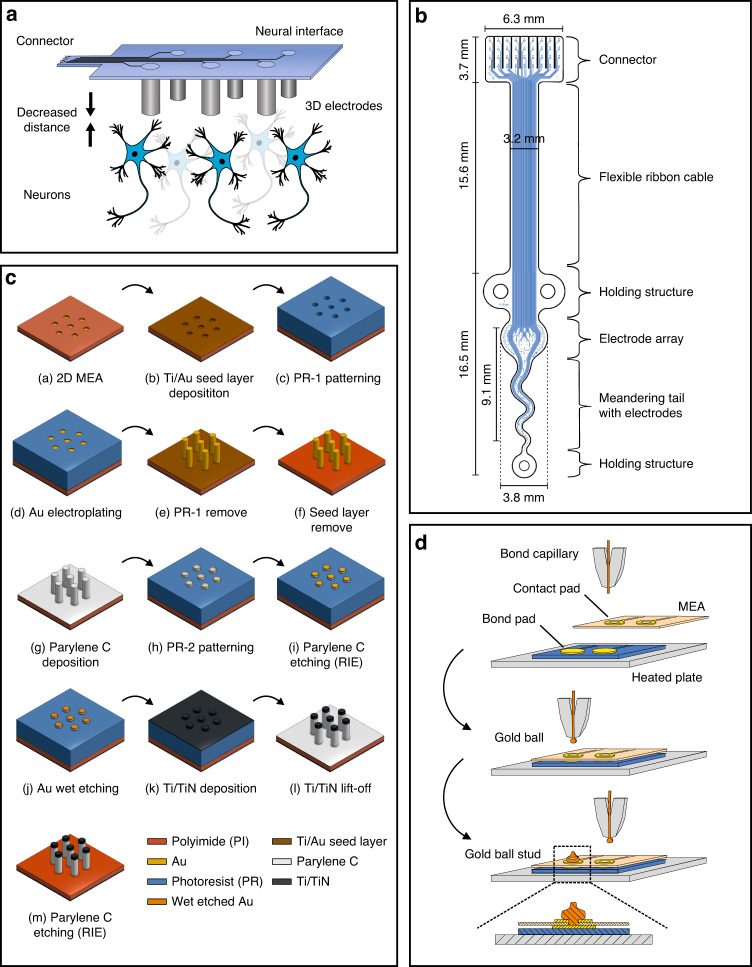
Design and fabrication process of the 3D neural interface. **a** Sketch of the neural interface with protruding microelectrodes decreasing the distance to the neurons. **b** Layout of neural interface design with dimensions and compartment descriptions. **c** Process flow for the microfabrication of a flexible polyimide-based 3D MEA. **d** Schematic view of the MFI technique connecting a flexible MEA with a rigid printed circuit board (PCB). A tiny gold ball (~Ø 90 µm) is formed at the capillary by the flame-off electrode. The gold ball is pushed against the hole of the MEA contact pad onto the heated PCB bond pad with a defined force and ultrasonic energy connecting the components mechanically and electrically. Finally, the capillary is lifted, and the gold wire is cut off, leaving a tiny gold stud that acts as a microrivet between the MEA and the PCB

Autonomic nerves and nerve plexuses represent the biological target areas for the developed neural interface. These small nerves mainly consist of unmyelinated C-fibers with inherently low neural-signal amplitudes. The distance between the microelectrodes and the neurons may be decreased due to the protruding nature of 3D electrodes so that even low-amplitude neural signals can be recorded (Fig. 2a).

The design of the thin-film neural interface (Fig. 2b) is divided into different functional sections to be optimally suited for the measurement of neural activity in the pelvic cavity. The finger structure design of the connector simplifies encapsulation and connection to an external adapter, which allows the neural interface to be used in electrophysiological experiments. A flexible ribbon mechanically decouples the electrode array from the measuring system. Ring-shaped structures at the tip and sides of the MEA facilitate handling and fixation during surgery. Small oval holes (35 · 70 µm²) distributed across the neural interface prevent air entrapment between the neural interface and the tissue. The measurement area of the MEA comprises two sections with different geometrical shapes. The first part allows for spatial measurements on plexus areas, while the second part is designed as a meandering tail to measure along the vagus/hypogastric nerves where space is limited.

The neural interface has 36 electrodes in total, including two reference electrodes and two ground electrodes (Fig. 2c). It was fabricated in two versions with electrode diameters of 20 or 50 µm.

### Cleanroom fabrication of the initial 2D thin-film MEA

Using standard microelectromechanical systems (MEMS) processes, a flexible polyimide-based MEA with gold conductive tracks and 2D gold electrodes was produced as the initial substrate (for details, see Fig. S1 in the Supplementary Information).

### Fabrication of cylindrical 3D gold microelectrodes

3D MEAs were fabricated by the electrodeposition of gold pillars on the initial 2D MEAs. Therefore, a starter layer of titanium (~50 nm) and gold (~100 nm) was sputtered (Z550, Leybold Heraeus GmbH, Koeln, Germany) onto the 2D substrate (Fig. 2c, step b) and patterned with a photoresist (AZ IPS-6050, MicroChemicals GmbH, Ulm, Germany), resulting in a 70 µm-thick template for the electrodeposition of gold pillars (Fig. 2c, step c).

The sample was hydrophilized with O_2_ plasma (PlasmaLab 800, Oxford Instruments, Abingdon, England) and cleaned by cyclic voltammetry (CV). A three-electrode setup was used with the substrate as the working electrode (WE), a platinated Ti mesh as the counter electrode (CE) and an Ag/AgCl (3 M KCl) reference electrode (RE) (for details, see Fig. S2 in the Supplementary Information). CV was performed at 21 °C in 0.5 M H_2_SO_4_ solution (H_2_SO_4_, 96%, MicroChemicals GmbH) with a potentiostat (Keithley 2450 Source Meter, Keithley Instruments, Cleveland, Ohio, USA) (15 cycles, 0.4–1.5 V vs. RE, scan rate of 100 mV/s). To remove the remaining air bubbles from the microstructures, the sample was treated with ultrasound (power input of 12 kJ) directly in the H_2_SO_4_ solution using a sonotrode (Sonopuls Mini20, BANDELIN electronic GmbH & Co. KG, Berlin, Germany).

The wafer was rinsed with deionized water (DIW), immersed in a gold electrolyte (NB Semiplate Au 100 AS, MicroChemicals GmbH) and again treated with ultrasound (15 kJ). The pH value of the electrolyte bath was set to 9.35. The electrolyte was constantly stirred at 120 rpm and kept at 33 °C. The electrodeposition of gold was carried out by chronoamperometry (Keithley 2450 Source Meter, Keithley Instruments) at a constant voltage of −550 mV vs. Ag/AgCl (3 M KCl) for 24 h to obtain gold pillars with a height of 60 µm. The deposition rate was calculated to be ~2.5 µm/h (current ~2 mA) (Fig. 2c, step d). The photoresist was stripped according to the manufacturer’s protocol (Fig. 2c, step e). The seed layer was removed by RIE (Z550, Leybold Heraeus GmbH) using Ar/CH_4_ plasma (Fig. 2c, step f).

### Sidewall insulation of the 3D microelectrodes with parylene C and pillar head modification with titanium nitride (TiN)

The substrate was coated with ~4 µm parylene C (DPX-C, Specialty Coating Systems, Indianapolis, IN, USA) at a process pressure of 3 Pa in a parylene coater (Comelec, La Chaux-de-Fonds, Switzerland). The sample was pretreated with Ar/O_2_ plasma and an adhesion promoter (methacryloxypropyltrimethoxysilane; Silane A-174, Merck, Darmstadt, Germany) (Fig. 2c, step g).

**Fig. 3:**
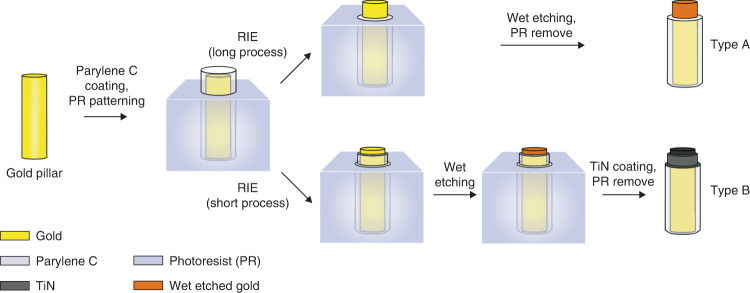
Different gold pillar modification methods. Type A: Gold pillar side-insulated with parylene C and wet etched. Type B: Gold pillar side-insulated with parylene C, wet etched, and coated with TiN

To modify the pillar heads, a photoresist (AZ IPS-6050, MicroChemicals GmbH) was applied onto the substrate according to the manufacturer’s protocol. The heads were opened by flood exposure (without a photomask) and development; the exposed head height was increased by longer exposure. To expose the heads to a height of 10 µm on 60 µm-high pillars, the exposure dose was 85 mJ/cm² (Fig. 2c, step h). Parylene C was removed from the exposed pillar heads by reactive ion etching (RIE) with O_2_ plasma (25 min, etch rate 12 µm/h) (PL800, Oxford Instruments) (Fig. 2c, step i). The exposed gold surface was roughened by wet etching (TechniEtch ACI2, MicroChemicals GmbH) for 2 min (Fig. 2c, step j). The pillar heads were coated with Ti/TiN (~500 nm) by sputter deposition (L-560, Leybold Heraeus GmbH) (Fig. 2c, step k) with Ti as an adhesion promoter (~50 nm). Ti/TiN lift-off was performed in acetone for 1 h (Fig. 2c, step l). Finally, parylene C, which remained on the planar regions of the PI substrate, was etched by RIE using O_2_ for 18 min (Fig. 2c, step m) to prevent substrate curling caused by internal mechanical stress. The planar substrate thickness of the final neural interface was ~9 µm. Electrodes manufactured according to the described process chain are referred to as “type B” (Fig. 3). Additionally, microelectrodes were fabricated that were not coated with TiN but only wet etched (referred to as “type A”, Fig. 3). For the type A electrodes, the head modification height was defined by the RIE duration (Fig. 2c, step i).

To evaluate the effect of surface roughening on the subsequent TiN coating, some samples were not wet etched but only coated with TiN (referred to as “type C”). The process chain and further results regarding this modification type are shown in the Supplementary Information, Fig. S3 and Fig. S6.

### Connection to external measurement equipment

**Fig. 4:**
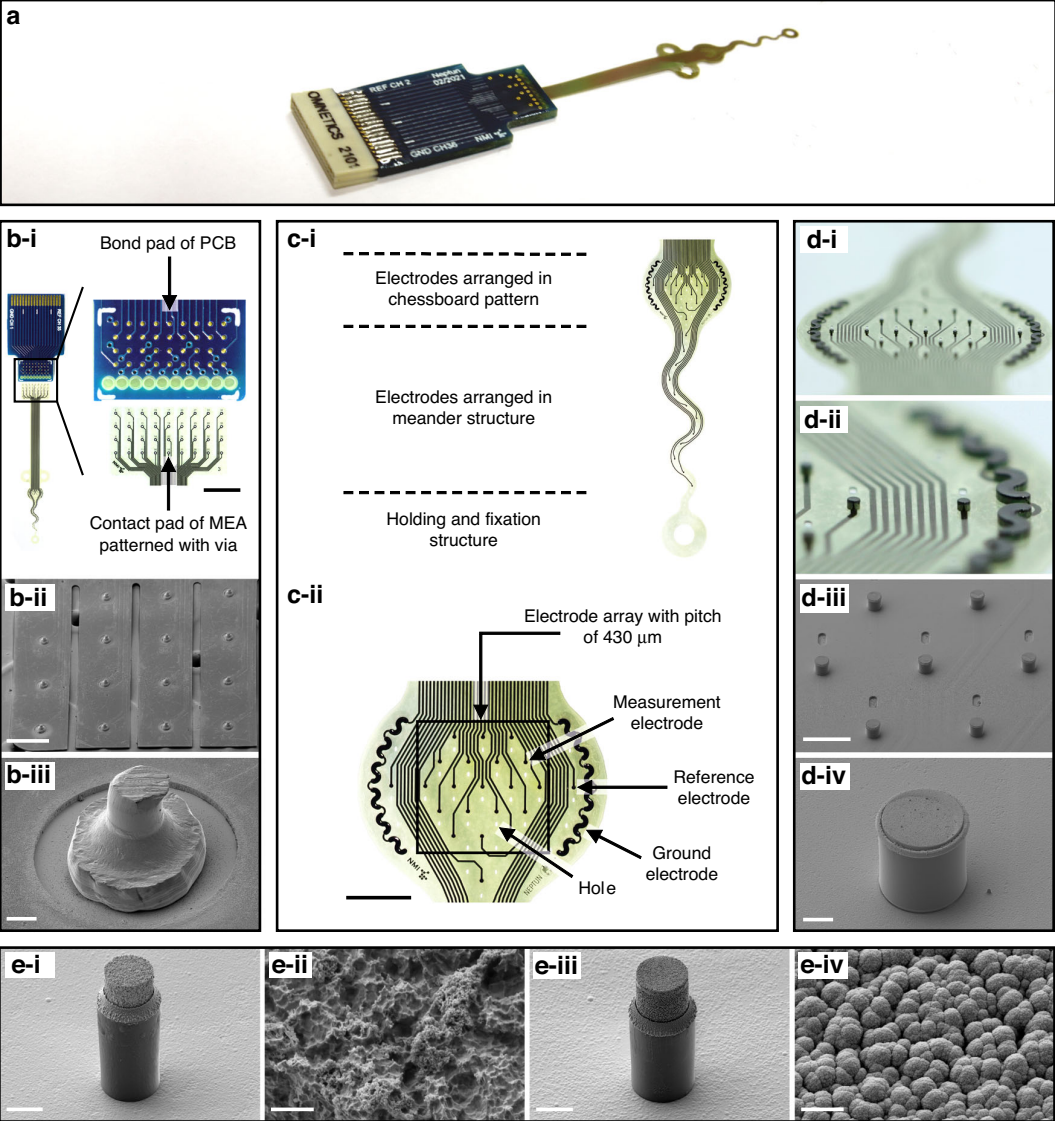
Fabricated 3D neural interface. **a** Photograph of the fabricated neural device showing the flexible MEA with 3D microelectrodes connected to a PCB with a soldered Omnetics connector. **b** Assembling of the neural interface. **b-i** Macroscopic image of the MEA connector with the corresponding PCB. **b-ii** Scanning electron microscopy (SEM) image of an assembled neural device with finger structures electrically connected by microrivets to the underlying PCB. **b-iii** SEM image of a single gold bump stud connecting the neural interface contact pad to the PCB bond pad. The entire hole is filled and covered by the gold stud, which acts as a microrivet. **c** Macroscopic images of the neural interface with compartment descriptions and functions of the entire measurement field (**c-i**) and of the area with electrodes arranged in a chessboard pattern (**c-ii**). The electrode heads were coated with TiN, which appears black in the images. **d** Macroscopic images (**d-i**, **d-ii**) and SEM images (**d-iii**, **d-iv**) of an MEA and of a single microelectrode (Ø 50 µm, height ~50 µm). The pillars exhibit parylene C side insulation, and their heads were wet etched and coated with TiN. The microelectrode shown in **d-iv** has a TiN coating on its head of 4–5 µm height. **e** SEM images of single modified microelectrodes, which were either wet etched (**e-i**, **e-ii**) or wet etched and TiN-coated (**e-iii**, **e-iv**) (both Ø 20 µm with a height of ~60 µm). The pillars were etched by RIE (Fig. 2c, step i) until the parylene C was removed from the exposed tops and sides of the pillar heads. Scale bars: **b**-**i**: 2 mm, **c**-**ii**: 1 mm; **b**-**ii**: 500 µm; **d**-**iii**: 200 µm; **b**-**iii**, **d**-**iv**, **e**-**i**, **e**-**iii**: 20 µm; **e**-**ii**, **e**-**iv**: 1 µm

MEAs were connected to printed circuit boards (PCBs) by a process known as the microflex interconnection technique (MFI)^55^ using a wire bonding device (Series 5610, F&S Bondtec Semiconductor GmbH, Braunau am Inn, Austria) equipped with a 25 µm-thick gold wire. The process required via holes (Ø 85 µm) in the flexible contact pads (Ø 135 µm) of the MEA (Fig. 4b). In the process, each MEA contact pad was connected with a PCB bond pad by a gold microrivet (Fig. 2d). For bonding, the ultrasound parameters were 100 digits of power and 30 ms. The force was set to 30 cN, and the stage temperature to 150 °C.

To mechanically stabilize and insulate the connection between MEA and PCB, an epoxy adhesive (Polytec EP 653, Polytec PT GmbH, Hoersching, Austria) was applied in the cavities (Ø 500 µm) of the connection pad array and cured according to the manufacturer’s protocol. An Omnetics connector (A79022, Omnetics Connector Corp. Minneapolis, MN, USA) was soldered to the PCB contact pads. The contact pads and bonding area were sealed with an epoxy adhesive (UHU Endfest Plus 300, UHU Holding GmbH, Buehl, Germany). A fabricated MEA, including the adapter PCB, is shown in Fig. 4a.

### Electrical characterization of the neural interface

**Fig. 5:**
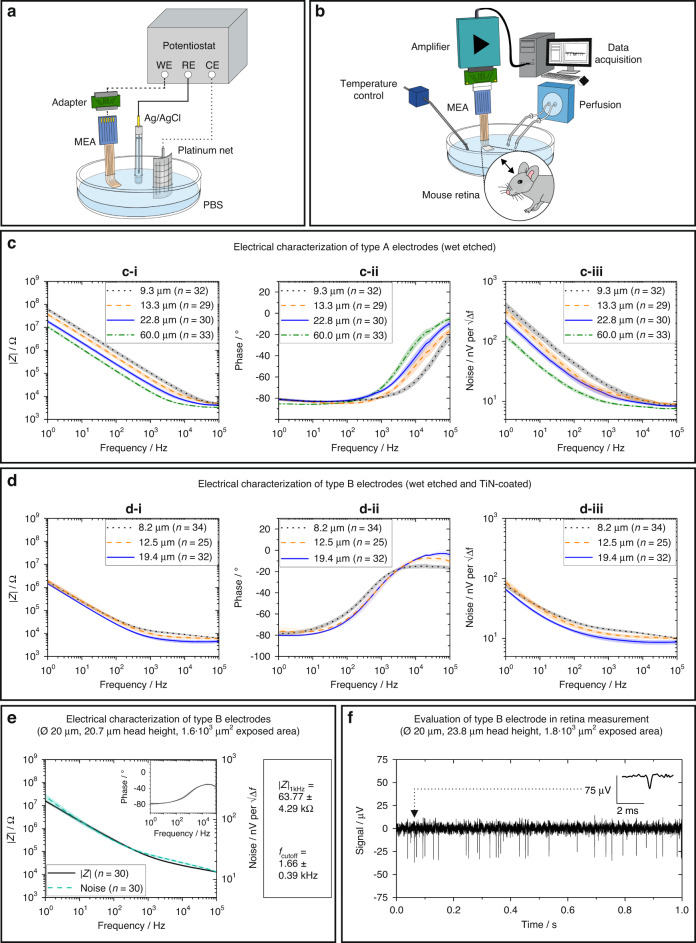
Electrical characterization of the fabricated 3D microelectrodes and performance of the developed neural interface in ex vivo experiments with mouse retinae. **a** Schematic of a three-electrode electrochemical system with the neural interface as the working electrode (WE), a platinum net as the counter electrode (CE) and an Ag/AgCl (3 M KCl) electrode as the reference electrode (RE). **b** Ex vivo experimental description of spike activity recordings on mouse retinae using the fabricated neural device. **c**, **d** Electrical in vitro characterization of different electrodes with a pillar diameter of 50 µm and a pillar height of 60 µm. For each sample, the electrode electrical impedance magnitude |Z| and phase were measured by applying a voltage of 100 mV and sweeping the frequency from 1 Hz to 100 kHz. The noise was calculated from the real part Re(Z) of the impedance Z. The impedance magnitude, phase and noise were averaged for each sample over the electrode number *n*. **c-i**, **c-ii**, **c-iii** Electrical characterization of gold electrodes encapsulated with parylene C, opened by RIE to a specific height and wet etched (Fig. 3, type A). The averaged impedance magnitude, phase and noise of electrodes with head heights of 9.3 µm (black), 13.3 µm (orange), 22.8 µm (blue), and 60.0 µm (green) are shown in individual diagrams, with the respective standard deviation indicated as the shaded area. **d-i**, **d-ii**, **d-iii** Electrical characterization of gold electrodes encapsulated with parylene C, opened by RIE, wet etched and coated with TiN to a specific height (Fig. 3, type B). The averaged impedance magnitude, phase and noise of electrodes with head heights of 8.2 µm (black), 12.5 µm (orange), and 19.4 µm (blue) are shown in individual diagrams, with the respective standard deviation indicated as the shaded area. **e** Electrical characterization of type B electrodes with a diameter of 20 µm and a 20.7 µm high head (with an overall height of 60 µm). The averaged impedance magnitude (black) and noise (light blue) are shown in a single diagram, with the averaged phase displayed in the right-hand corner. The corresponding values for |Z|_1kHz_ and *f*_cutoff_ are given in the box on the right side of the diagram. **f** Recording of retinae spike activity with a type B electrode of diameter 20 µm with an exposed head of 23.8 µm height. A magnification of a single spike is shown in the top right corner of the diagram

3D MEAs were electrically characterized by electrochemical impedance spectroscopy (EIS) with an impedance/gain phase analyzer (Gamry Instruments Inc., Warminster, PA, USA). EIS was performed in phosphate-buffered saline (PBS) solution (PBS 10010023, Thermo Fisher Scientific, Waltham, MA, USA) at 21 °C using a three-electrode setup with the MEA as the WE, a platinum net as the CE and an Ag/AgCl (3 M KCl) electrode as the RE (Fig. 5a). Prior to the measurement, microelectrodes were hydrophilized with an air plasma for 90 s and soaked in PBS for 1 h. The samples were contacted with a customized PCB allowing the electrode channels to be measured automatically one after another. A sinusoidal AC input voltage of 100 mV was applied, and the frequency was swept from 1 Hz to 100 kHz while measuring the impedance.

For each modification type, at least three samples were characterized that comprised electrodes with a diameter of 50 µm and a height of 60 µm. The heads were modified to have dimensions between 8.2 and 60 µm in height. In addition, an MEA comprising type B electrodes with a diameter of 20 µm was characterized. At least 25 microelectrodes were measured for each sample.

The thermal or Johnson noise ν_n_ of a microelectrode can provide information about its suitability for recording electrophysiological data. A theoretical estimation for the Johnson noise ν_n_ over a frequency band ∆f can be derived from the real part Re(Z) of the measured impedance spectrum according to Eq. (1)^56,57^:

1 $$\nu _n = \sqrt {4 \cdot k_{\mathrm{B}} \cdot T \cdot {\mathrm{Re}}\left( {\underline Z } \right) \cdot \Delta f}$$

where *k*_B_ = 1.38 · 10^−23^ J/K is the Boltzmann constant, and *T* = 310 K is the absolute temperature of the human body. For each measured sample, the noise was plotted as ${\nu _n}/{\sqrt {\Delta f} }$. Integrating ν_n_ over a defined frequency bandwidth yields the electrode’s noise in volts for the electrode in a respective measurement.

### Performance of the neural interface in mouse retinae experiments (ex vivo)

All experimental procedures were carried out in compliance with the institutional guidelines of the NMI and approved by the Regierungspräsidium Tübingen according to German Federal laws on animal welfare.

Retinal preparation was performed using adult B6.CXB1-Pde6brd10/J (*rd10*) mice of either sex as described previously^58–61^. The age of the *rd10* mice varied between 400–430 days postnatal.

The mice were anesthetized and euthanized by cervical dislocation. After enucleating and hemisecting the eyes, the retinae were peeled off the sclera and dissected in Ames’ medium (Merck KGaA, Darmstadt, Germany).

Prior to the experiment, the 3D MEA was hydrophilized with air plasma (Plasma Cleaner/Sterilizer PDC-32G, Harrick Plasma Inc., NY, USA) for 30 s and coated with ~500 µl (1 mg/ml) poly-l-lysine (P1399 MW, 150–300 kDa, Merck KGaA) to improve the adhesion of the retinae to the neural interface.

Before placing the retina on the microelectrodes, the 3D MEA was rinsed with Ames’ solution. Retina portions (~2 ・ 2 mm^2^) were prepared under a dissecting microscope and placed in an epiretinal configuration onto the 3D MEA such that the retinal ganglion cells (RGCs) and microelectrodes were in close proximity. The 3D MEA with the attached retina portion was constantly perfused with warm and oxygenated Ames’ medium (flow rate 2–4 ml/min, temperature 32–35 °C).

The neural interface with the retina portion was connected to a ME2100 electrophysiology system (Multi Channel Systems MCS GmbH, Reutlingen, Germany) using the ME2100-HS32-M headstage (Fig. 5b). The recordings of retinal signals were low-pass filtered (10 kHz) and digitally sampled (25 kHz, 24 bit). To reduce perfusion-pump artifacts, we applied software filtering (30 Hz high-pass). The amplifier was grounded to reduce interference (for details, see Fig. S4 in the Supplementary Information). After each experiment, the 3D MEAs were cleaned with Terg-a-zyme (Merck KGaA) dissolved in bidistilled water. Retina measurements were performed using MEAs with type B electrodes.

## Results

### Optical characterization of the 3D microelectrodes

The 3D microelectrode arrays with 36 microelectrodes were fabricated and fully assembled (Fig. 4). MEAs were reliably connected to PCBs with high accuracy using the MFI method (Fig. 4b). Concerning the electrode design, 19 electrodes were distributed over the envisioned recording area in a chessboard pattern with a spacing of 430 µm and 13 electrodes were arranged along a meandering tail (Fig. 4c-i). A reference electrode with the same geometry as the measuring electrodes and a meandering ground electrode with 500 times the base area of a measuring electrode were located on each side outside the array (Fig. 4c-ii).

Gold pillars with dimensions of 20 µm as well as 50 µm in diameter and 60 µm in height (Fig. 4d, e) were electroplated with a success rate of up to 100% by adjusting the process parameters. The height of the pillars was limited by the template photoresist thickness (max. ~90 µm) (Fig. 2c, step e). To avoid the electrodeposition of pillars with mushroom shapes, the pillar height was chosen to be less than the template thickness.

Pillar sides were successfully insulated with parylene C, and the heads were opened by RIE with the exposed head height controlled by the etching duration.

As previously described, exposed pillar heads were further modified by either wet etching of gold (type A) or wet etching of gold and additional TiN coating (type B) (Fig. 4di, d-ii). The degree of pillar head modification could be adjusted in a reproducible process with µm accuracy, which allowed the fabrication of pillars with customized head heights (see Fig. S5 in the Supplementary Information). The photoresist mask for modifying the pillar heads was very homogeneous in thickness, which made it possible to uniformly modify the electrodes of an entire MEA, even those with low head heights (Fig. 4d-iii, iv). After modification, the parylene C insulation was still intact.

The micromachining of pillar heads increased the surface roughness compared to that of the unmodified gold pillars. Wet etching produced a porous coral-like surface (Fig. 4e-ii), while the subsequent TiN coating resulted in fractal surface morphology (Fig. 4e-iv).

Overall, the process technology presented in this work allowed the specific tailoring of the electrical properties of the microelectrodes by adjusting the size of the exposed pillar head surface and the coating material.

### Electrical characterization of the 3D microelectrodes

The impedance and noise of type A and type B electrodes (Fig. 3) were analyzed and compared. Four samples of electrodes with gold interfaces (Fig. 5c-i, -ii, -iii) and parylene C sidewall insulation at different heights (type A) all followed the same tendency. In the high-frequency range, all four samples had a similar impedance magnitude, which was ~2–5 kΩ. The impedance steadily increased with decreasing frequency.

The overall impedance spectra were shifted according to the size of the exposed surface area of the respective microelectrodes. Electrodes with a 60 µm head had the lowest impedance magnitude of 15.82 ± 1.68 kΩ at 1 kHz, while the electrodes with a 9.3 µm head had the highest impedance magnitude of 104.15 ± 18.36 kΩ. All type A samples showed a predominantly capacitive behavior with a phase angle approaching −90°. In the higher frequency range, the phase angle increased, which is characteristic of a rather resistive behavior.

An estimation of the filter characteristics of the microelectrodes can be obtained from the respective phase diagrams by extracting the cutoff frequency *f*_cutoff_. The frequency corresponding to a phase angle of −45° indicates *f*_cutoff_, which provides information about the signal band in which frequency-dependent filtering takes place at the interfacial boundary. Signals above *f*_cutoff_ are reproduced by the recording electrode in their unmodified form, while signals below *f*_cutoff_ are nonlinearly distorted. The transfer properties of electrodes act as high-pass filters. *f*_cutoff_ represents the transition to the pass-band, which is called the access resistance regime^27,28^. Estimating *f*_cutoff_ can therefore be helpful for evaluating the filtering and recording properties of microelectrodes.

For the type A microelectrodes, *f*_cutoff_ decreased with increasing surface area. For electrodes with a 9.3 µm head, *f*_cutoff_ was ~31.51 ± 4.96 kHz, while for electrodes with a 60 µm head, it was only 4.34 ± 0.81 kHz, indicating that these microelectrodes represented measured signals more authentically over a broader bandwidth. In agreement with the impedance spectra, the calculated Johnson noise followed a similar dependence on the surface area for the four MEAs having type A electrodes. The electrode with the largest surface area clearly exhibited the lowest noise.

The results of the TiN-coated electrode sites (Fig. 5d-i, -ii, -iii)) followed the same tendencies but had lower cutoff frequencies and overall reduced impedances.

The access resistance of all type B electrodes was between 3–6 kΩ at 100 kHz, where the phase angle was between 0° and −20°. The impedance remained almost constant for the respective electrodes up to a frequency of 1 kHz and rose significantly in the low-frequency range. As in the case of the type A electrodes, a well-known dependency between the surface size and the impedance magnitude was found^28,31,62^. At 1 kHz, the electrodes with the largest surface area (19.4 µm head height) had an average impedance magnitude of approx. 6.78 ± 0.89 kΩ, whereas the electrodes with the smallest surface area (8.2 µm head height) had an average impedance magnitude of ~13.19 ± 1.17 kΩ.

The phase angles of all type B microelectrodes showed a predominantly resistive behavior in the high-frequency range, which became capacitive in the low-frequency range approaching ~−80°. For electrodes with a TiN-coated head of 19.4 and 12.5 µm height, *f*_cutoff_ was very similar (0.49 ± 0.18 and 0.48 ± 0.09 kHz), while it decreased for the electrodes with an 8.2 µm TiN head (0.30 ± 0.04 kHz). All type B electrodes with a diameter of 50 µm therefore authentically reproduced measured signals down to less than 0.5 kHz, which was more than eight times lower than the *f*_cutoff_ of a type A electrode with a comparable diameter and a head height of 60 µm.

The noise of the TiN-coated microelectrodes followed a similar trend as the corresponding impedance. The microelectrodes with the highest surface area showed the lowest noise over the complete bandwidth of 1 Hz to 100 kHz. Electrodes with an 8.2 µm head had the highest noise, which was only lower than the noise of the electrodes with a 12.5 µm head below a frequency of 10 Hz.

While the access resistances of type A and type B microelectrodes were comparable, the impedance magnitude and Johnson noise were significantly lower for all type B samples over the entire frequency band. In Table 1, the calculated exposed surface area A, the average impedance magnitude |Z|_1kHz_ at 1 kHz and the cutoff frequency *f*_cutoff_ are summarized for all samples.

**Table 1:** Summary of electrical characterization data for measured microelectrodes.

Type	Exposed pillar height in µm	Exposed surface area (A) in 10^3^ µm²	|Z|_1kHz_ ± SD in kΩ	*f*_cutoff_ ± SD in kHz	No. of electrodes (*n*)
A	9.3	3.4	104.15 ± 18.36	31.51 ± 4.96	32
A	13.3	4.1	57.54 ± 10.81	13.25 ± 2.96	29
A	22.8	5.5	32.56 ± 5.35	8.36 ± 1.90	30
A	60.0	11.4	15.82 ± 1.68	4.34 ± 0.81	33
B	8.2	3.3	13.19 ± 1.17	0.30 ± 0.04	34
B	12.5	3.9	9.85 ± 1.32	0.48 ± 0.09	25
B	19.4	5.0	6.78 ± 0.89	0.49 ± 0.18	32

Type A pillars were wet etched where their surface area was not coated with photoresist (defined as exposed area A); type B pillars were wet etched and subsequently coated with TiN. All microelectrodes were side-insulated with parylene C (except for type A electrodes with a head height of 60 µm). The pillars had a diameter of 50 µm and an overall height of ~60 µm. The values for the exposed pillar head, the exposed electrode area (A), the average impedance magnitude at 1 kHz (|Z|_1kHz_) and the cutoff frequency (*f*_cutoff_) are listed for all samples.

For the type B electrodes, pillars with a diameter of 50 µm and additionally pillars with a diameter of 20 µm were characterized (Fig. 5e). Measured electrodes with a head height of 20.7 µm exhibited the smallest exposed surface area (1.6 · 10^3^ µm²) in comparison to all characterized electrodes. The impedance, phase and noise curves followed the same tendencies that were observed for the type B electrodes with a diameter of 50 µm. With a value of 63.77 ± 4.29 kΩ, the electrodes with a diameter of 20 µm showed the highest impedance magnitude at 1 kHz for all type B electrodes. The cutoff frequency *f*_cutoff_ was also the highest for the respective type (1.66 ± 0.39 kHz) but lower in comparison to the *f*_cutoff_ of all type A electrodes.

The type C electrodes showed a very similar electrical behavior to the type B electrodes. The corresponding EIS data are provided in Fig. S6 and Table S1 in the Supplementary Information.

### Performance of the neural interface in mouse retina experiments (ex vivo)

By conducting a pilot study, it was possible to measure spontaneous spike activity using our flexible 3D microelectrode array on explanted mouse retinae. Recordings were taken with a neural interface with type B microelectrodes with a diameter of 20 µm and a head height of 23.8 µm. Spontaneous retinal spikes were clearly identified on several channels up to a height of −37 µV (Fig. 5f). The single spikes had a duration of less than 1 ms.

The recorded spiking activity of the retina was in the pass-band of the presented 20 µm-diameter type B electrode with a cutoff frequency of approximately 1.7 kHz (Fig. 5).

Type A electrodes were not selected for the mouse retina experiments because they revealed overall poorer electrical properties in the characterization study.

As the electrical properties of type C electrodes were similar to those of type B electrodes, retina measurements were also performed with type C electrodes, and spikes up to −66 µV were detected using electrodes with a diameter of 50 µm and a head height of 12.4 µm (for more information, see Fig. S6 in the Supplementary Information).

## Discussion

### Evaluation of process technology for fabricating a neural interface with 3D microelectrodes

Several approaches for the fabrication of 3D MEAs have been reported in the literature to study neuronal networks with the aim of advancing the vision of patient-individualized bioelectronic medicine^63–65^. Of particular relevance is the development of protruding microelectrodes that reduce the distance to the neurons, thus improving signal quality^33,66^. 3D microstructures can be fabricated by several methods, such as deep reactive ion etching (DRIE)^17,67^, micromolding^68,69^ or electrodeposition combined with lithographic processes^33,70–72^.

For example, Spanu et al.^73^ developed a 3D MEA with passivated gold pillars for brain-on-a-dish applications by the electrodeposition of gold on top of a standard glass 2D MEA (pillar Ø 60–65 µm, height ~110 µm). A similar approach was chosen in this work to fabricate a flexible neural interface with pillars (Ø 20 and 50 µm, height ~60 µm), which were micromachined with TiN and parylene C. Electrodeposition can be easily combined with MEMS technologies. Unlike DRIE, which inherently requires a solid base material, such as silicon, electrodeposition is compatible with various substrate materials, including polymers, which expands its range of applications^53^.

As introduced earlier, Yan et al.^52^ combined DRIE with silicone casting and backside alignment to fabricate a Utah-like stretchable 3D MEA. However, the process flow involves complex steps, which usually affect fabrication costs and device design.

Electrodeposition, however, is a cost-effective, simple technology. Nevertheless, it is essential to monitor the electrolyte since its composition slightly changes in concentration during plating. Furthermore, the plating properties are influenced by the electrode geometry, pattern configuration and electrolyte hydrodynamics^70^.

Within our study, we confirm that the quality and success of electrodeposition were dependent on the aspect ratio of the pillars, and the parameters had to be adjusted. It is well known that surface tension can be a threshold for wetting small-diameter openings and depends on the hydrophilicity of the materials involved^74^. To successfully plate electrodes with a diameter of 20 µm and height of 60 µm, ultrasonic and plasma pretreatments were necessary to completely wet the template openings during photoresist development as well as before starting the electroplating process (for more details, see Fig. S7 in the Supplementary Material).

As an outlook, the stability of microelectrodes could be improved while maintaining their selectivity by shaping large-diameter pillars (e.g., >50 µm) into cones and modifying their tips after deposition.

### Optical and electrical characterization of the 3D microelectrodes

There is still great interest in exploring innovative electrode materials to further improve the electrical and mechanical properties as well as the biocompatibility of neural interfaces. Miniaturized electrodes with diameters of 4–100 µm allow the recording of single-unit activity with high spatial resolution^75^. However, the smaller the surface area of the electrode is, the higher its impedance and also, therefore, its inherent Johnson noise.

Modifying electrodes with sputtered iridium oxide films (SIROFs), TiN, nanostructured platinum (nanoPt) or conductive polymers, such as poly(3,4-ethylenedioxy-thiophene) (PEDOT), increases an electrode’s effective surface area and therefore decreases its impedance and noise^29,71,76^. In addition, these materials provide optimized electrode characteristics, such as higher electrochemical conductivity and a higher charge injection capacity, compared to those of classical metals, such as gold, platinum, or stainless steel^27^. Regarding stimulation, the use of low-impedance electrodes allows the delivery of sufficient current to activate excitable tissue at lower potentials, thereby reducing the risk of electrochemical side effects.

The results of this paper are in accordance with the state-of-the-art knowledge that the roughness of the interface material, which determines the electrochemical surface area, prevails over the underlying substrate properties in materials such as TiN and Pt, in which no volume effects (e.g., PEDOT) or valence changes (e.g., IrO_x_) could be expected^30–32,76,77^.

Coating the rough gold electrodes with TiN significantly decreased the electrode impedance and, thus also, the thermal noise. Moreover, the decreased cutoff frequency suggests that TiN-coated electrodes record signals more authentically over a broader frequency band.

The electrode sidewalls were passivated with parylene C, which is known for its high biocompatibility, chemical and biological inertness, and long-term stability^53,67^. Although parylene C is reported to exhibit poor adhesion on gold^78^, the EIS measurements, as well as the SEM images of the passivated and etched pillars, support the assumption of sufficient adhesion between the polymer and gold. The passivated pillar sidewalls did not show a morphology change after being exposed to the gold etching solution. Thus, the wet etching process was also proof of quality for the completeness of the parylene C removal from the pillar heads.

Parylene C can be conformally deposited as a thin-film passivation layer with a thickness in the micrometer range and still provides good barrier properties. In this work, it was well suited as a sidewall insulating material for electrodes, but its limitations in terms of film thickness became apparent as the 4 µm-thick coating increased the 20 µm pillar diameter by 40%. As the development of miniaturized structures progresses to the nanoscale, further innovative passivation coatings need to be considered. Thin-film metal oxide coatings applied by Atomic Layer Deposition (ALD) are gaining attention as ultrathin conformal coatings with high barrier properties^79–81^.

### Performance of the flexible protruding microelectrode array

The results of the ex vivo retina pilot study present a proof of concept for the measurement of the physiological action potentials of RGCs with the fabricated 3D neural interface.

The microelectrode noise during the measurements was low enough to clearly identify defined single spikes of ~1 ms in duration.

The condition of the retina patch, the preparation technique and the contact between neuronal tissue and electrode mainly determine the signal quality, and therefore, the preparation itself has a deep impact on the number, shape and amplitude of the detected signals. These experiments are not suitable for electrode characterization but demonstrate that the fabricated electrodes can be used to measure local neuronal potentials in principle.

Several groups have already demonstrated that the subretinal implantation of a retinal prosthesis with 3D protruding electrodes increased its proximity to RGCs and therefore improved the efficiency of the implant device^33,82,83^. Since RGCs form the first layer of the retina directly facing the neural interface and the axons in the retinal nerve fiber layer are unmyelinated^84,85^, the retina represents a valuable system for measuring neuronal action potentials with high signal quality.

Autonomic neuronal structures, however, are small peripheral nerves composed of unmyelinated C-fibers. For example, Agostoni et al. reported that the feline abdominal vagus nerve consists of ~30,000 fibers composed of 88% unmyelinated fibers with a thickness of less than 6 µm^86^. Peripheral nerves are bundled in superordinate structures, each surrounded by different protective layers, making the selective measurement of neuronal signals difficult. Regarding stimulation, a high current is required to activate the inherently small C-fibers^87^. Since unmyelinated fibers have very low conduction velocities that vary with fiber diameter, the already small signal broadens with increasing distance from a stimulation site^88^.

Taking these considerations in mind, recording evoked sum potentials from autonomic nerves with the 3D MEA is assumed to be more challenging than detecting the single-unit activities of RGCs. A comparison of the measurement results is therefore only possible to a limited extent. Nevertheless, several groups have reported the successful recording of signals from autonomic nerves^17,89–91^. For example, Payne et al.^92^ measured the evoked C-fiber response of the abdominal vagus nerve in rats using a bipolar platinum cuff electrode. The reported electrode surface area was 60 times higher with only slightly lower impedance compared to that of the modified electrodes (type B, 19.4 µm TiN head height) presented in this work.

We, therefore, propose the fabricated 3D neural interface as a potential tool for measuring neural signals from pelvic neural structures.

As an outlook, in vivo acute and chronic recordings need to be conducted to further characterize the performance and long-term stability of the 3D neural interface. In addition, postmortem histological investigations could provide information on the incorporation behavior of the microelectrodes and their biocompatibility.

In summary, we presented a proof of concept study, including device development and validation in an ex vivo setup. The process technology enabled the reproducible fabrication of a polyimide-based 3D neural interface with modified microelectrodes exhibiting improved recording properties, such as a low electrical impedance and noise. The protruding microelectrodes allowed recording closer to the signal source and within the target tissue, thus improving the signal quality. We demonstrated that combining electrodeposition with standard lithographic processes opens up a range of possibilities for specifically tailoring microelectrode properties, such as geometry and mechanical and electrical characteristics. The thin-film neural interface with 3D microelectrodes can be implemented in a variety of areas, ranging from in vitro cell culture or tissue preparations to in vivo recordings of the central or peripheral nervous system.

## Supplementary information

Supplemental Material

## Data Availability

The data that support the findings of this study are available from the corresponding author upon reasonable request.
