# Supplementary material for: A flexible protruding microelectrode array for neural interfacing in bioelectronic medicine
Source: Microsyst Nanoeng. 2022 Dec 22;8:131. doi: 10.1038/s41378-022-00466-z (PMC9772315; doi:10.1038/s41378-022-00466-z)
Supplement: Supplementary file 1 — Supplemental Material [file 41378_2022_466_MOESM1_ESM.pdf]

Supplementary Information

S1. Cleanroom fabrication of the initial 2D thin-film microelectrode array

For the fabrication of the 2D MEAs, a first PI layer (PI-2611, HDMicrosystems, Parlin, NJ, USA) with a 6 µm thickness was spin-coated onto a one-side polished 4-inch silicon handling wafer (**Figure S1**, *step a*). To enhance the adhesion to the subsequent metallization layer, the PI was roughened by oxygen plasma (Z550, Leybold Heraeus GmbH, Koeln, Germany) and coated with a silicon oxide layer using hexamethyldisiloxane (HMDSO) (abcr GmbH, Karlsruhe, Germany) as a precursor in a plasma-enhanced chemical vapor deposition (PECVD) process. A combination of Ti, Au and Ti was deposited on PI using a sputtering process (Z550, Leybold Heraeus GmbH) (**Figure S1**, *step b*). Ti and Au were deposited with thicknesses of 50 nm and 300 nm, respectively, with Ti serving as the adhesion layer to the PI and Au being the metal layer for the conduction lines and electrodes. The metallization was patterned by a photoresist (AZ ECI 3027, MicroChemicals GmbH, Ulm, Germany) and dry etched (Z550, Leybold Heraeus GmbH) (**Figure S1**, *step c*). After the removal of the photoresist, a second layer of PI (~6 µm) was spin-coated onto the substrate (**Figure S1**, *step d*), followed by the deposition of a silicon nitride hard mask by PECVD (PlasmaLab 800, Oxford Instruments, Abingdon, England) (**Figure S1**, *step e*). The hard mask, which served as a mask to structure the PI, was patterned using the same photoresist and was then etched by RIE using CF_4 (PL800, Oxford Instruments). After structuring the hard mask, the photoresist was removed, and PI was etched with O_2 plasma by RIE (PL800, Oxford Instruments). Subsequently, CF_4/O_2 plasma was applied to remove the hard mask, followed by a soft RIE process with CF_4 (PL800, Oxford Instruments) to etch the remaining Ti/TiO from the gold electrodes (**Figure S1**, *step f*).

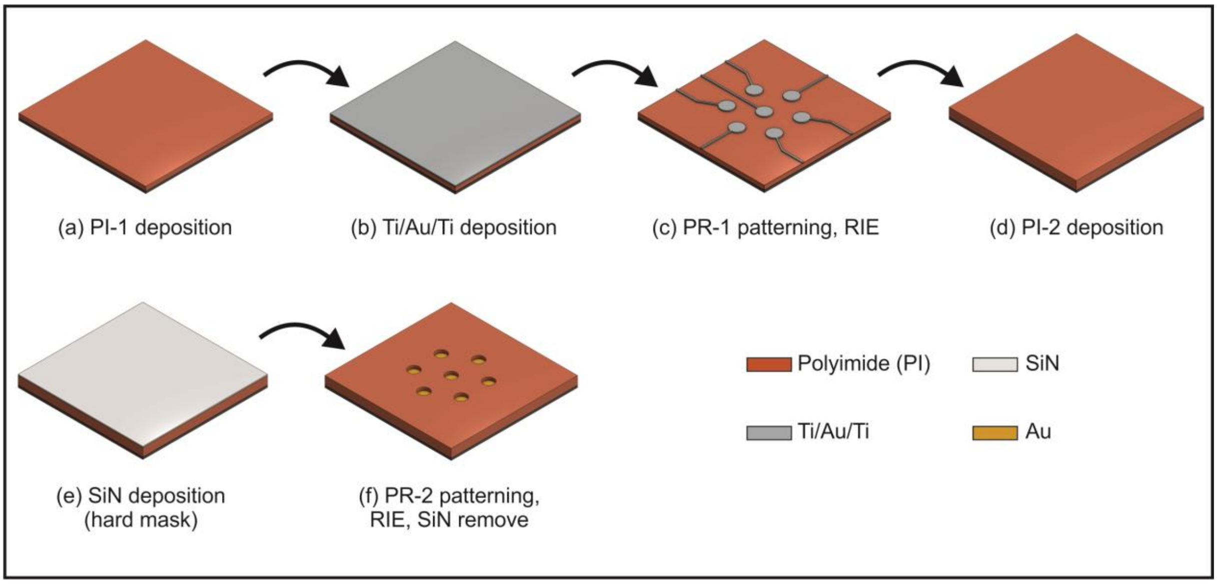

Figure S1: Process flow for the microfabrication of a flexible polyimide-based 2D microelectrode array.

S2. Electroplating setup

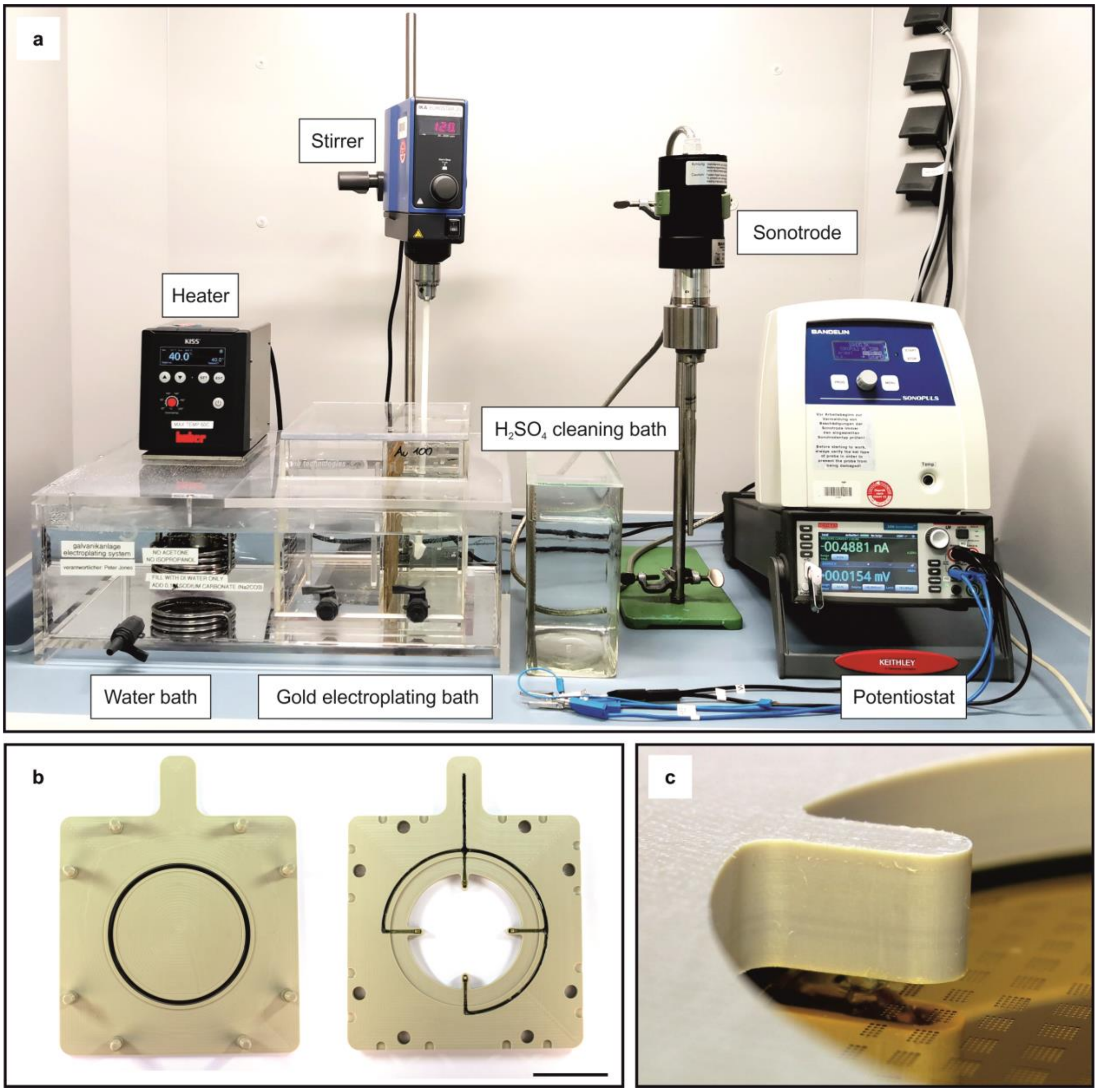

Figure S2: Electroplating setup. **a** Photograph of the electroplating setup. **b** Electroplating holder. The wafer can be clamped between the two holding parts onto the sealing rings and securely fixed by screws. During clamping, four electrical spring contacts are pressed against the contact pads of the wafer to establish the electrical connection between the sample and the potentiostat. Scale bar: 5 cm. **c** Close-up of electrical spring contact pushing on the seed layer of the sample.

S3. Fabrication guide of type C electrodes (not wet etched)

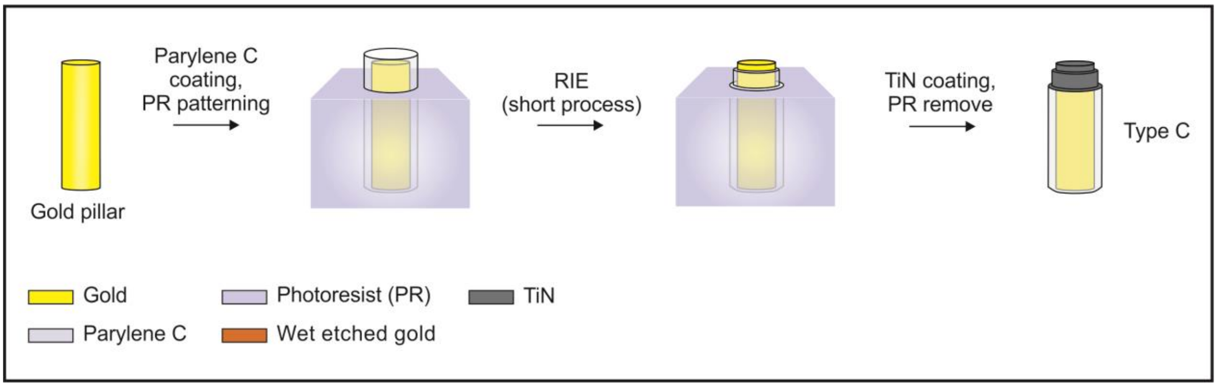

Figure S3: Gold pillar modification guide for type C electrodes. Type C electrodes were side-insulated with parylene C, opened by RIE and coated with TiN.

S4. Measurement setup of mouse retina experiments

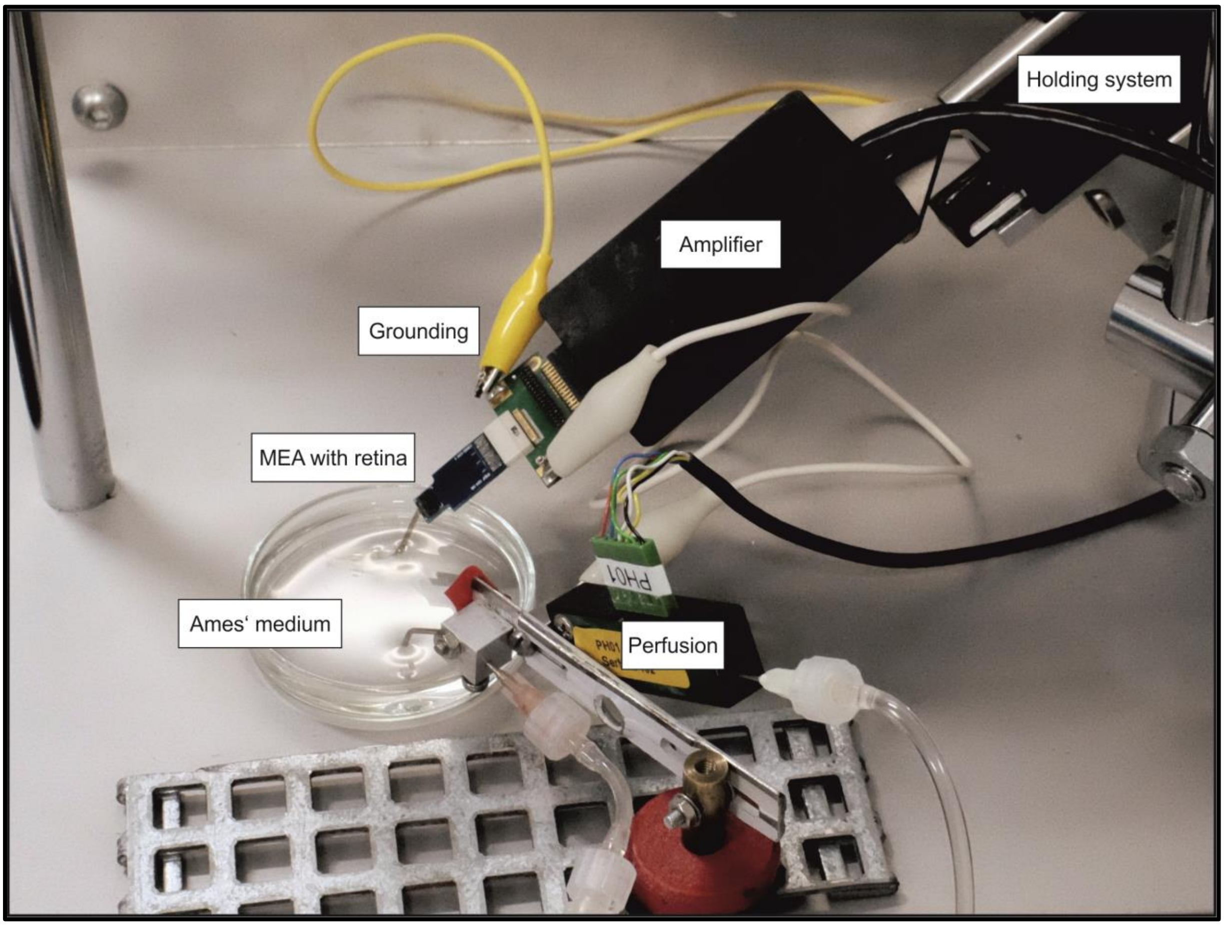

Figure S4: Measurement setup of the mouse retina experiments. The MEA with the attached retina portion was continuously perfused with warm and oxygenated Ames' solution in the petri dish. The connection between the MEA and ME2100 headstage was established via a green PCB adapter, which was grounded. The temperature control (not shown) was removed during the measurement.

S5. Macroscopic images of differently modified 3D microelectrodes

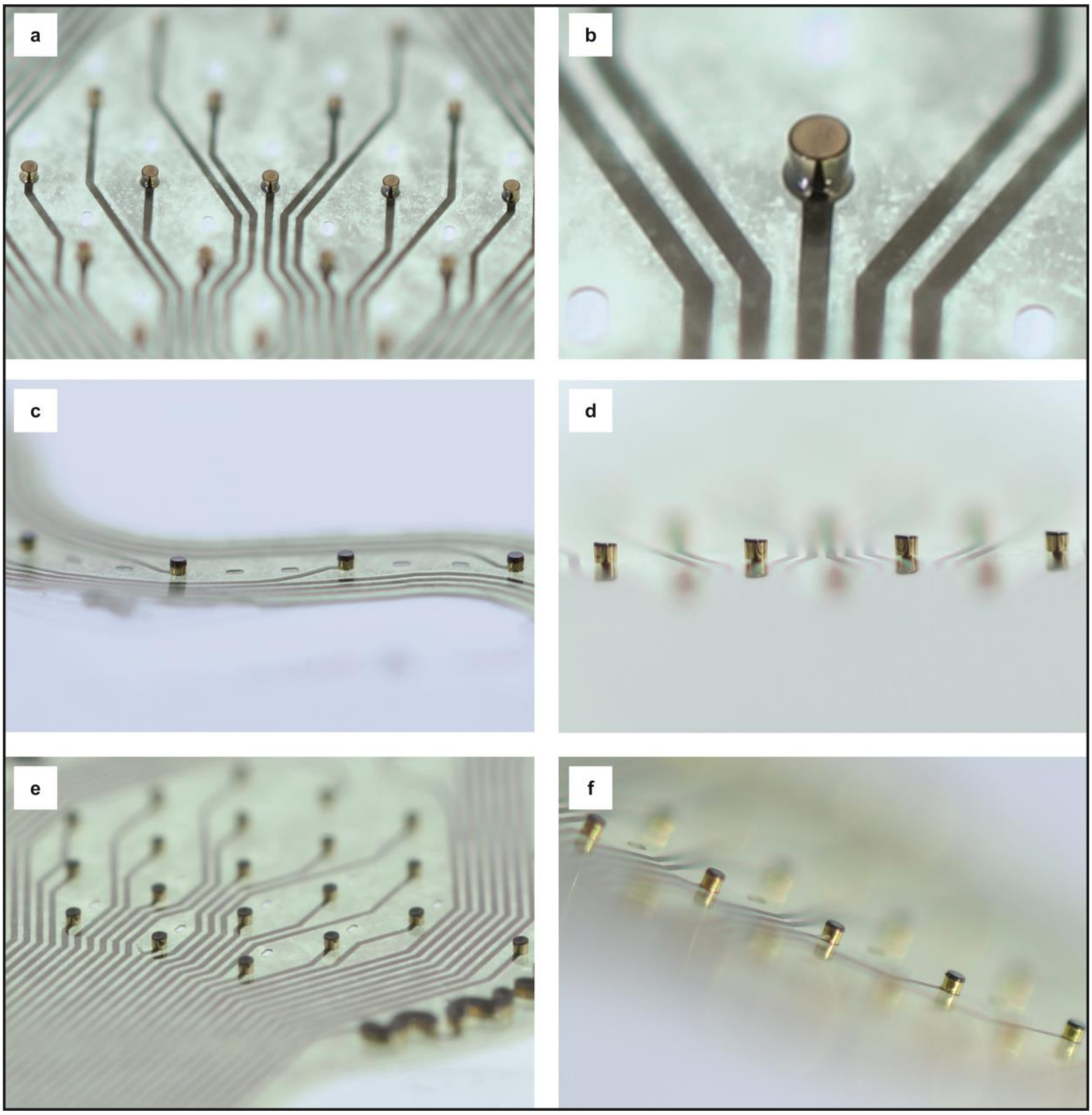

Figure S5: Macroscopic images of modified pillars. a, b Wet etched pillars that were opened at their heads by RIE. **c** TiN-coated pillars (17.8 µm head height). **d** TiN-coated pillars (8.5 µm head height). **e, f** Wet etched and TiN-coated pillars (12 µm head height). All pillars had a diameter of 50 µm, a pillar height of 60 µm and were side-passivated with parylene C.

S6. Electrical characterization and performance evaluation of type C electrodes (not wet etched)

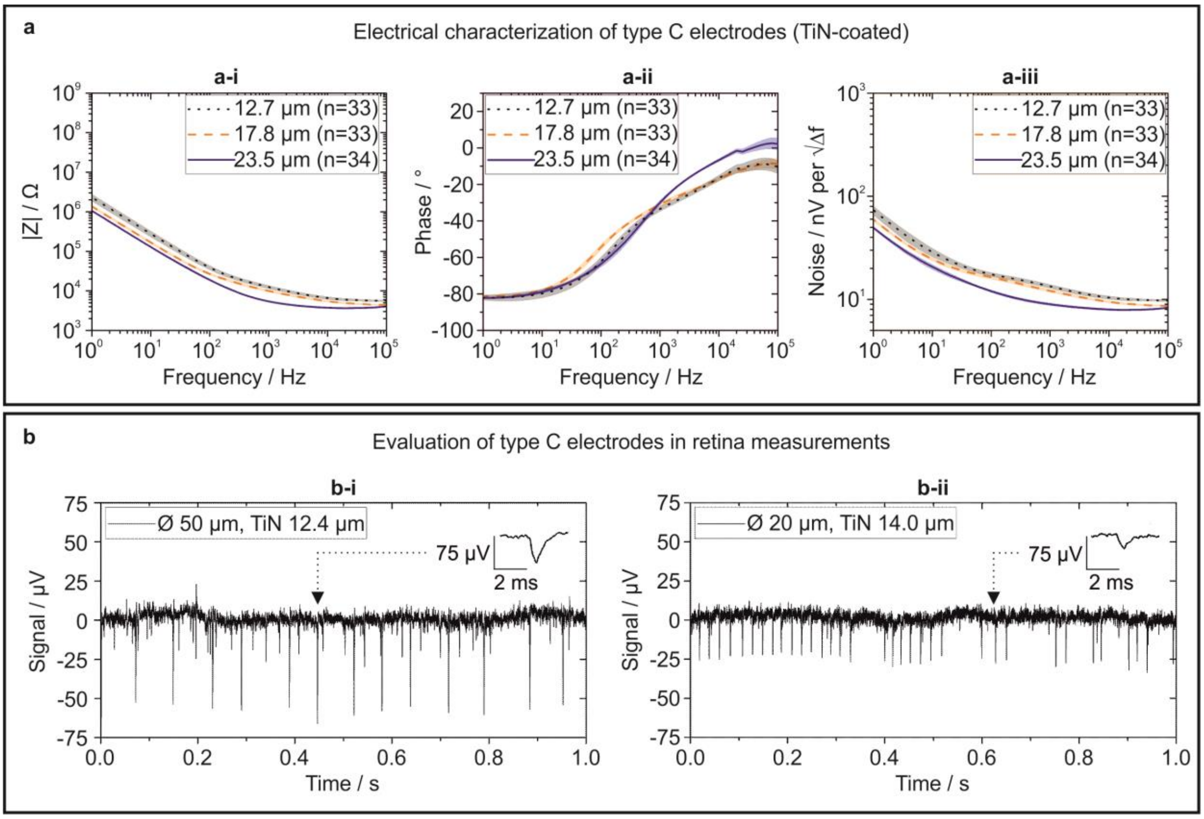

Figure S6: Electrical characterization of type C microelectrodes and evaluation in ex vivo retina experiments. a Electrical in vitro characterization of gold electrodes that were encapsulated with parylene C, opened by RIE to a specific height and coated with TiN. All electrodes had a pillar diameter of 50 µm and a pillar height of 60 µm. For each sample, the electrode electrical impedance magnitude |Z| and phase were measured by applying a voltage of 100 mV and sweeping the frequency from 1 Hz to 100 kHz. The noise was calculated from the real part Re($\underline{Z}$) of the impedance Z. The impedance magnitude, phase and noise were averaged for each sample over the electrode number n. The averaged impedance magnitude **(a-i)**, phase **(a-ii)** and noise **(a-iii)** of electrodes with a head height of 12.7 µm (black), 17.8 µm (orange) and 23.5 µm (blue) are shown in individual diagrams with the respective standard deviation indicated as shaded area. **b** Recordings of retinae spike activity with type C electrodes. For each measurement, a magnification of a single spike is shown. **b-i** Measurement of spike activity with a neural interface with Ø 50 µm type C electrodes (TiN-coated heads with a height of 12.4 µm and an exposed surface area of $3.9 \cdot 10^3$ µm²). **b-ii** Measurement of spike activity with a neural interface with Ø 20 µm type C electrodes (TiN-coated heads with a height of 14.0 µm and an exposed surface area of $1.2 \cdot 10^3$ µm²).

Table S1: Summary of electrical characterization data for measured type C microelectrodes. Type C pillars were insulated with parylene C and opened by RIE to a defined height. The exposed head with surface area A was subsequently coated with TiN. The pillars had a diameter of 50 µm and an overall height of ~60 µm. The values for the exposed pillar head, the exposed surface area (A), average impedance magnitude at 1 kHz ($|Z|_{1kHz}$) and the cutoff frequency (f_{cutoff}) are listed below for all measured samples.

Type	Exposed pillar height in µm	Exposed surface area (A) in 10^3 µm²	$|Z|_{1kHz}$ ± SD in kΩ	f_{cutoff} ± SD in kHz	No. of electrodes (n)
C	12.7	3.96	12.53 ± 1.18	0.31 ± 0.04	33
C	17.8	4.80	9.97 ± 0.63	0.21 ± 0.02	33
C	23.5	5.65	5.42 ± 0.25	0.39 ± 0.07	34

S7. SEM images of the electrodeposition experiments

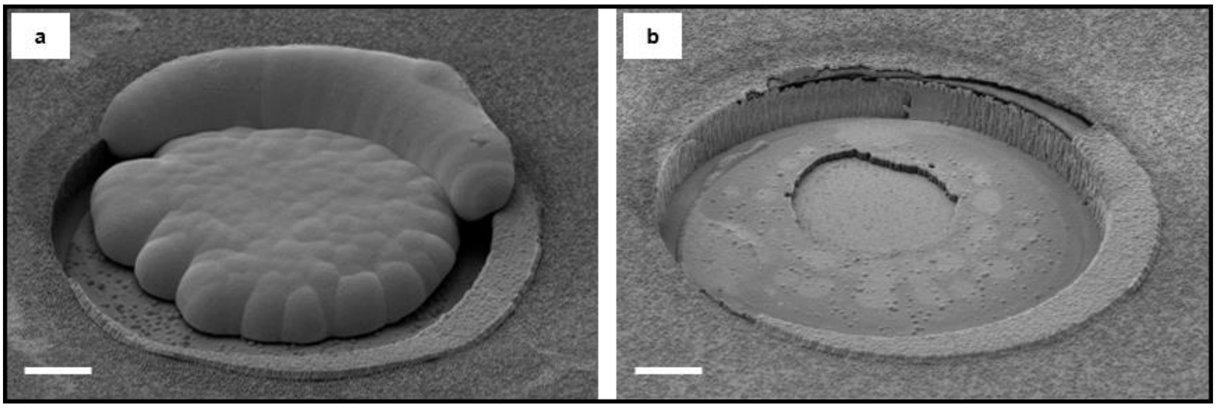

Figure S7: SEM images of the failed growth of electroplated pillars due to photoresist residues in the template holes. **a** SEM image of a 2D electrode on which gold grew only on areas that were free of photoresist. The nucleation sites gradually grew together. **b** Pillar that detached from its base along with the seed layer, leaving a small cavity. Bright spots indicate places where the base was free of photoresist and gold was electroplated onto the seed layer. Scale bar: 5 µm.
